# DNA Damage Checkpoint Responses in the S Phase of Synchronized Diploid Human Fibroblasts

**DOI:** 10.1111/php.12361

**Published:** 2014-11-24

**Authors:** Paul D Chastain, Bruna P Brylawski, Yingchun C Zhou, Shangbang Rao, Haitao Chu, Joseph G Ibrahim, William K Kaufmann, Marila Cordeiro-Stone

**Affiliations:** 1 College of Osteopathic Medicine, William Carey UniversityHattiesburg, MS; 2 Department of Pathology and Laboratory Medicine, University of North CarolinaChapel Hill, NC; 3 Lineberger Comprehensive Cancer Center, University of North CarolinaChapel Hill, NC; 4 Department of Biostatistics, University of North CarolinaChapel Hill, NC; 5 Division of Biostatistics, School of Public Health, University of MinnesotaMinneapolis, MN; 6 Center for Environmental Health and Susceptibility, University of North CarolinaChapel Hill, NC

## Abstract

We investigated the hypothesis that the strength of the activation of the intra-S DNA damage checkpoint varies within the S phase. Synchronized diploid human fibroblasts were exposed to either 0 or 2.5 J m^−2^ UVC in early, mid- and late-S phase. The endpoints measured were the following: (1) radio-resistant DNA synthesis (RDS), (2) induction of Chk1 phosphorylation, (3) initiation of new replicons and (4) length of replication tracks synthesized after irradiation. RDS analysis showed that global DNA synthesis was inhibited by approximately the same extent (30 ± 12%), regardless of when during S phase the fibroblasts were exposed to UVC. Western blot analysis revealed that the UVC-induced phosphorylation of checkpoint kinase 1 (Chk1) on serine 345 was high in early and mid S but 10-fold lower in late S. DNA fiber immunostaining studies indicated that the replication fork displacement rate decreased in irradiated cells at the three time points examined; however, replicon initiation was inhibited strongly in early and mid S, but this response was attenuated in late S. These results suggest that the intra-S checkpoint activated by UVC-induced DNA damage is not as robust toward the end of S phase in its inhibition of the latest firing origins in human fibroblasts.

## Introduction

Eukaryotic cells have evolved a complex network of molecular reactions that work in concert to decrease the genotoxic effects of DNA damage. This network includes the activation of pathways to repair or tolerate DNA damage and checkpoints that slow cell cycle progression. During DNA synthesis (S phase), the intra-S checkpoint delays replication at the initiation and elongation steps and contributes to the stability of replication forks stalled at template lesions 1,2. DNA damaging agents that generate double-stranded breaks, such as ionizing radiation, are thought to activate the intra-S checkpoint initially *via* the ATM-Chk2-Cdc25A signaling pathway, whereas agents that produce DNA adducts, such as methyl methanesulfonate, benzo(a)pyrene diol epoxide and ultraviolet light, activate primarily the ATR-Chk1-Cdc7-Dbf4 pathway [reviewed in 3,4]. Regardless of which intra-S checkpoint arm becomes activated, one of the end results is that replication initiation is inhibited. Inhibition of origin firing can be abrogated by eliminating or reducing the abundance of one of the proteins associated with checkpoint activation or by adding an inhibitor of one of the checkpoint proteins [reviewed in 3]. In addition, replication forks progress more slowly in cells exposed to a DNA damaging agent than sham-treated cells 5–7. This reduced fork displacement rate is thought to be a combination of both passive and active mechanisms; some replication forks stall upon encountering template lesions (passive inhibition), while the rate of progression of others is actively reduced by a yet undefined intra-S checkpoint-mediated signaling mechanism that depends upon Tipin (Timeless-interacting protein), Hus1, Chk1 and XRCC3 (X-ray repair complementing defective in Chinese hamster cells 3) 6,8–12.

Although much has been discovered about how S phase cells in an asynchronous population respond to DNA damage, little is known about how vertebrate cells respond when challenged at different times in the S phase. In *Saccharomyces cerevisiae*, two mechanisms are used to slow S phase upon DNA damage by methyl methanesulfonate: inhibition of origin initiation by the Mec1-Rad53 intra-S checkpoint pathway and stalling of replication forks 13,14. Late-firing origins are preferentially inhibited after DNA damage, and the progression of replication forks is passively inhibited (i.e. upon encountering DNA damage) 13,14. In *Saccharomyces pombe,* the same two mechanisms delay S phase progression, but instead of late-firing origins being inhibited, as in *S. cerevisiae*, early firing origins are partially delayed, and late origins continue to fire 15. In addition, replication forks appear to be inhibited not only by encountering DNA damage but also by an active mechanism.

The goal of this study was to characterize the ability of the ATR-Chk1 checkpoint to become activated at different time points within the S phase of normal human fibroblasts (NHF) exposed to ultraviolet light (UVC). Our data indicate that normal human fibroblasts seem to behave more similarly to *S. pombe* than *S. cerevisiae*. In early and mid S phase, DNA synthesis in irradiated cells decreased through the inhibition of replication fork progression and origin initiation. Late in S phase, UVC-induced Chk1 phosphorylation (an indicator of ATR activation) and inhibition of origin initiation were attenuated. Interestingly, the replication fork rate was still reduced.

## Materials and Methods

### Cell lines and culture conditions

Normal human fibroblast strains 1 and 10 (NHF1 and NHF10, respectively) were derived from the foreskins of healthy babies 16,17 and immortalized by ectopic expression of the catalytic subunit of human telomerase 18. The cells were maintained in log phase in minimum Eagle's medium (Invitrogen) supplemented with 2 mm glutamine (Invitrogen) and 10% fetal bovine serum (Sigma) at 37°C in a humidified atmosphere of 5% CO_2_.

### UVC irradiation

Before exposure to UVC, the culture medium was removed from the plates and reserved. The plates were washed once with warm phosphate buffered saline (PBS) and placed uncovered under a UV lamp emitting primarily 254-nm radiation (UVC) at a fluence rate of 0.7 J m^−2^ s^−1^. Following irradiation, the reserved medium was added back, and the cultures were incubated for the indicated period of time. Sham-treated cultures were handled exactly the same way, except they were not exposed to UVC.

### Synchronization

For experiments requiring synchronization with aphidicolin (AG Scientific, San Diego, CA, USA), the cells were prepared as described previously 19,20. Briefly, the cells were grown to confluence to arrest them in G_0_ and replated at a lower density to allow the cells to re-enter the cell cycle in the presence of aphidicolin (2 *μ*g mL^−1^); the cultures were incubated for 24 h to accumulate the cycling cells at the beginning of the S phase 19–21. The DNA synthesis inhibitor was removed from the cells by washing the cultures 3 times with warm Hanks’ balanced salt solution (HBSS, HyClone) and adding pre-warmed fresh culture medium. The cells were exposed to UVC at 45 min, 3 h and 5 h after release. For synchronization without the inhibitor, the cells were grown to confluence arrest, replated at a lower density to allow them to re-enter the cell cycle, and treated 15, 18, 21 and 24 h after replating.

### Flow cytometry

The cells for flow cytometric analysis were labeled for 30 min with 30 *μ*m 5-bromo-2-deoxyuridine (BrdU) starting 45 min, 3 and 5 h after their release from aphidicolin inhibition or 15, 18, 21 and 24 h after replating from confluence arrest in the absence of aphidicolin. The cells were trypsinized, collected, washed twice with cold PBS and fixed in 70% cold ethanol. The staining was performed as previously published 22 using either a FITC-conjugated anti-BrdU mouse antibody (Becton Dickinson) or BrdU mouse antibody (Becton Dickinson) followed by an anti-mouse FITC-conjugated secondary antibody (Sigma); propidium iodide staining was used to measure the DNA content. The data were collected and analyzed using a CyAn flow cytometer (Beckman Coulter, Indianapolis, IN, USA). To estimate the percentage of cells in S phase from each of the dot plots illustrated, boxes were drawn manually around the area of low propidium iodide – low FITC-BrdU staining (cells in G_o_/G1), and around the area of high propidium iodide – low FITC-BrdU staining (cells in G_2_); then, the area encompassing the dots with FITC-BrdU staining clearly above the other two areas identified the cells in S phase.

### Radioresistant DNA synthesis (RDS)

The cells were sham treated or exposed to 2.5 J m^−2^ UVC (triplicate plates) according to the protocol described above to determine the UV-induced inhibition of DNA synthesis at various times in S phase (45 min, 3 h and 5 h after release from aphidicolin inhibition). After irradiation, the reserved medium was added back to the plates, and the cells were incubated at 37°C for 30 min and then labeled with [^3^H]-thymidine (5–10 *μ*Ci mL^−1^) for the next 30 min. The radioactive medium was removed, and the cells were washed three times with PBS, followed by incubation in 5% trichloroacetic acid (TCA) at 4°C for at least 30 min. TCA was removed, and the plates were washed twice with cold 5% TCA and three times with 70% ethanol. The plates were dried in a chemical fume hood and stored at 4°C until further analysis. To measure the incorporated radioactivity, 3 mL of freshly prepared 0.3 m NaOH was added to each plate; after at least 30 min at 37°C, triplicate aliquots of each cell lysate were counted in a Packard scintillation counter in Ecolite+ (MB Biochemicals). The remaining cell lysate was used to read its absorbance at 260 nm in a Beckman DU640 spectrophotometer as an index of the cell count per sample to normalize the incorporated ^3^H radioactivity.

### Western immunoblot analysis

The cells were irradiated at 45 min, 3 h or 5 h after release from aphidicolin inhibition (or 15, 18, 21 and 24 h after replating from confluence arrest in the absence of aphidicolin) and incubated in reserved medium for 30 min at 37°C. The cells were harvested by trypsinization, washed once in PBS and resuspended in lysis buffer [50 mm Tris-HCl (pH 8.0), 5 mm EDTA, 100 mm NaCl, 0.5% NP-40 and 2 mm DTT, supplemented before use with 10 mm*β*-glycerophosphate, 1 mm sodium orthovanadate and protease inhibitor cocktail (Sigma Aldrich, St. Louis, MO, USA)]. The protein concentrations were determined using the Bio-Rad D_C_ protein assay. Samples containing equal amounts of protein were mixed with an equal volume of 2x Laemmli sample buffer [125 mm Tris-HCl (pH 6.8), 4% SDS, 20% glycerol] containing 5% *β*-mercaptoethanol, boiled for 5 min and separated by SDS-polyacrylamide gel electrophoresis (SDS-PAGE). The proteins were transferred to nitrocellulose and probed with antibodies against Chk1 (sc-8408) and Chk1 phosphorylated at Ser^345^ (sc-17922, Santa Cruz Biotechnology, Inc., Santa Cruz, CA, USA). The enhanced chemiluminescent signal [ECL or ECL+ (GE Healthcare)] was recorded on an X-ray film (GE Healthcare).

The relative level of phosphorylated Chk1 (Chk1-P) at each time point was determined by digitally imaging the immunoblot results captured on film and quantifying the density of specific protein bands using an Alpha-Innotech gel imaging system and AlphaEaseFC software. To compare and average the results from different experiments, the data were normalized as follows: for each image, the density values for all total Chk1 bands were averaged, and the signal in each band was expressed as the ratio of its density over the average. The same procedure was followed for the Chk1-P bands. The final signal intensity for each Chk1-P band was then expressed relative to the total Chk1 signal in the same sample (normalized Chk1-P signal divided by the normalized total Chk1 signal).

### DNA fiber spreading

The cells were released from aphidicolin arrest as described above, and 10 min prior to being 45 min, 3 h or 5 h into S, they were pulsed for 10 min with 10 *μ*m IdU. Then, the cultures were washed twice with warm PBS, sham treated or irradiated with 2.5 J m^−2^ of UVC and immediately pulsed with 100 *μ*m CldU for 20 min. After the second pulse, the cells were collected and processed for fiber spreading as described previously 7. The incorporated 5-iodo-2-deoxyuridine (IdU) and 5-chloro-2-deoxyuridine (CldU) were fluorescently detected using an antibody sandwich that labeled the IdU tracks red and the CldU tracks green 7,10,22,23.

### Statistical analysis

To estimate the effect of UVC (2.5 J m^−2^) on RDS and Chk1 phosphorylation, a generalized linear model with robust standard errors was used to model the relative CPM/A260 and Chk1-P/Total Chk1 ratios on a log-transformed scale 24. A nonlinear, mixed effect model was used to estimate the effect of UVC (2.5 J m^−2^) on the percentage of CldU-only tracks. A linear mixed effect model was used to model the track length on the log-transformed scale. A Wald-type test was used to determine the statistical significance. All of the statistical analyses were performed with SAS 9.2 (SAS Institute Inc., Cary, NC, USA).

## Results

**Figure 1:**
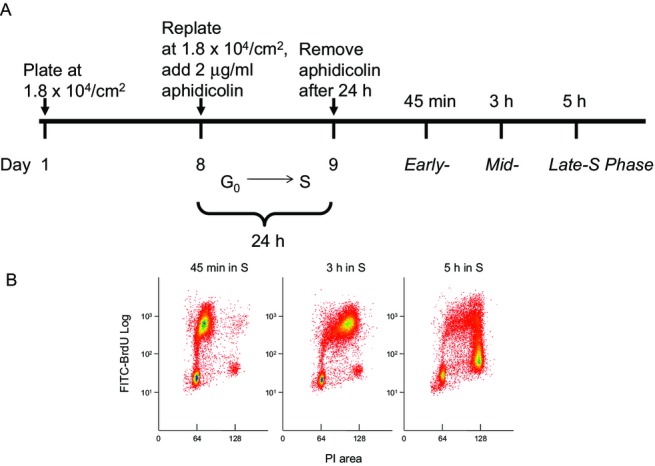
Aphidicolin treatment of cells released from confluence arrest improves the synchronized progression of human fibroblasts through the S phase. (A) Schematic of the synchronization protocol (not drawn to scale). (B) Bivariate flow cytometric analysis of NHF1-hTERT labeled with BrdU for 30 min starting 45 min, 3 h and 5 h into S; the DNA content was measured by propidium iodide staining. The percentage of cells in S at these time points was 70, 78 and 49%, respectively.

Normal human fibroblasts immortalized by expression of the catalytic subunit of human telomerase (NHF-hTERT) become quiescent at confluence and re-enter the cell cycle upon replating at a lower density. In the presence of aphidicolin, DNA synthesis is inhibited, and cells accumulate in early S phase. Once this temporary arrest is lifted by removal of the inhibitor, the cell population traverses the S phase with a higher degree of synchrony than that obtained with confluence arrest only. We used the synchronization protocol schematically illustrated in Fig.** **1A to obtain NHF1-hTERT populations enriched for early, mid- or late-S phase cells at 45 min, 3 h or 5 h, respectively, after removal of the DNA synthesis inhibitor (Fig.** **1B). The collection of enriched populations within specific windows of S made possible the investigation of potential variations in the magnitude of intra-S checkpoint responses in UVC-irradiated human fibroblasts.

### UVC inhibits DNA synthesis in cells synchronized to early, mid- and late-S phase

**Figure 2:**
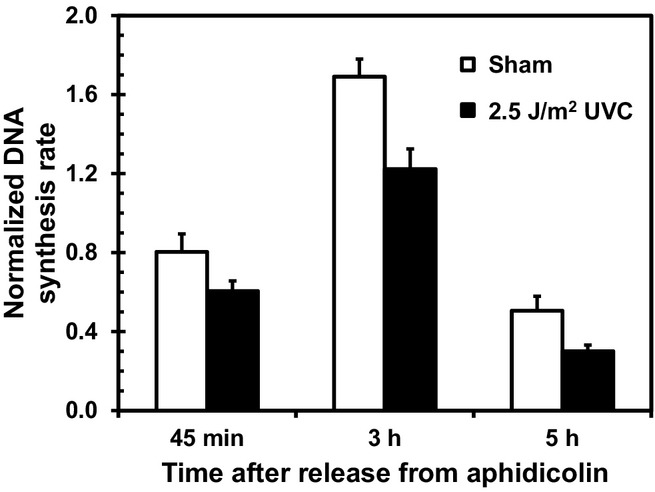
UVC inhibits DNA synthesis in cells synchronized to early, mid- or late-S phase. NHF1-hTERT cells were sham treated or irradiated with 2.5 J m^−2^ UVC at the indicated times and pulsed for 30 min with ^3^H-thymidine. The incorporation of ^3^H-thymidine into acid-insoluble macromolecules was normalized to absorbance at 260 nm (see Materials and Methods for details). Next, the relative CPM/A260 values were determined by dividing the normalized ^3^H-thymidine incorporation in each sample by the average CPM/A260 in the sham group within an experiment. The illustrated data represent averages of these calculated values (*n* = 4); the error bars indicate the standard error of the mean. Irradiation inhibited the overall DNA synthesis rate in early (*P* = 0.0006), mid (*P* < 0.0001) and late-S phase (*P* < 0.0001); the UVC-induced inhibition was 23 ± 15% in early S, 28 ± 8% in mid S and 39 ± 8% in late-S phase; no significant differences in the magnitude of this inhibition was found between early and mid S (*P* = 0.64), while significant differences were found between early and late S (*P* = 0.0013) and between mid and late S (*P* = 0.0055).

The overall rate of DNA synthesis in the synchronized populations varied as NHF1-hTERT progressed through S phase; after exposure to UVC, DNA synthesis was inhibited (on average, 30 ± 12%) at the three time points (Fig.** **2). This radiation-dependent inhibition should reflect both active responses (checkpoint activation) and the passive blockage of replication forks by template lesions. These parameters were investigated by pulse labeling the synchronized populations with fluorescent DNA precursors before and after UVC exposure and examining the DNA fiber staining patterns when the cells were in early, mid- or late-S phase.

### DNA damage inhibited replication fork progression throughout the S phase, but inhibition of origin activation was attenuated in late S

**Figure 3:**
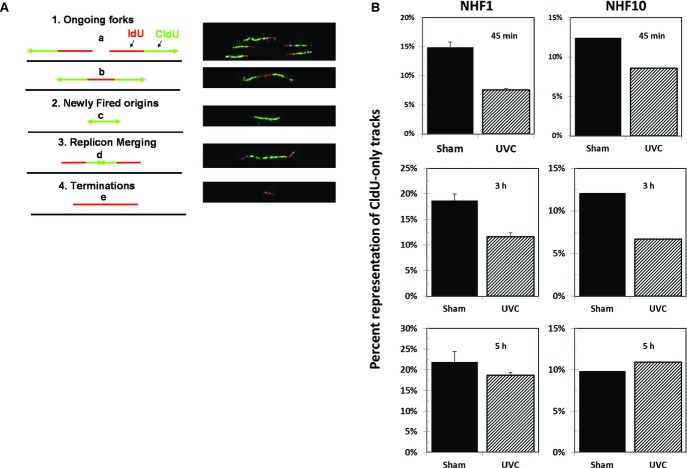
Cells late in S do not inhibit origin initiation after UVC exposure as efficiently as in early and mid S phase. (A) Left panel: Schematic illustration of the labeling patterns of active replication units before (IdU, red) and after (CldU, green) irradiation with 0 or 2.5 J m^−2^ of UVC. Right panel: Examples of replication units labeled through a 10-min pulse with IdU followed by a 20-min pulse with CldU; photomicrographs and schematic drawings of the corresponding fibers. (B) Percent of CldU-only replication tracks (new initiation) in NHF1-hTERT and NHF10-hTERT after treatment with 0 or 2.5 J m^−2^ UVC at 45 min, 3 h or 5 h into S phase. UVC exposure in NHF1-hTERT was associated with a strong inhibition of replicon initiation at 45 min (*P* = 0.0031) and 3 h (*P* = 0.0096), while an attenuated response was observed at 5 h (*P* = 0.0855); see Table1. The error bars represent the standard errors of the mean with *n* = 4. Results of a single experiment with NHF10-hTERT agreed well with those obtained with NHF1-hTERT.

DNA fiber analysis was performed in NHF1-hTERT synchronized by confluence/aphidicolin arrest (Fig.** **1), prepulsed with IdU for 10 min, exposed to 0 (sham) or 2.5 J m^−2^ UVC, and immediately pulsed with CldU for 20 min. Replication units that terminated before UVC exposure (or that were immediately arrested) were identified as red-only (IdU-only) (Fig.** **3A**,** pattern 4); replication units activated after treatment (i.e. during the second pulse) were identified as green-only (CldU-only) (Fig.** **3A, pattern 2); finally, replication units active before UVC exposure (IdU) and that continued to incorporate halogenated precursors (CldU) were identified as red-green (IdU-CldU) tracks (Fig.** **3A, patterns 1a, 1b and 3). The analysis of the distribution of scored replication tracks in these three categories (Table** **1) confirmed that exposure of S phase cells to 2.5 J m^−2^ UVC caused a decrease in CldU-only tracks (indicating inhibition of origin initiation). Interestingly, the origins that fired late in S seemed more resistant to inhibition in both NHF1-hTERT (Fig.** **3B) and in another immortalized normal human fibroblast cell line, NHF10-hTERT, than origins initiated in early and mid S phase.

**Table 1:** Distribution of tracks that incorporated only IdU (red-only), only CldU (green-only) or both labels (red-green) in synchronized NHF1-hTERT cells that were pulsed for 10 min with IdU, sham treated or irradiated with 2.5 J m^−2^ UVC light at 45 min, 3 h or 5 h in S, and then pulsed with CldU for 20 min. Aggregate data from four independent experiments are shown

	Red-Only (IdU) (%)	Green-Only (CldU) (%)	Red-Green (IdU-CldU) (%)	Total Scored
45 min				
SHAM	129 (8.9)	221 (15.2)	1105 (75.9)	1455
UVC	378 (24.5)	118 (7.6)	1049 (67.9)	1545
3 h				
SHAM	246 (20.9)	214 (18.2)	716 (60.9)	1176
UVC	348 (36.8)	111 (11.7)	486 (51.4)	945
5 h				
SHAM	224 (24.9)	203 (22.6)	473 (52.6)	900
UVC	405 (35.8)	213 (18.8)	514 (45.4)	1132

UVC treatment caused a significant overall effect on the percentage of green-only tracks (*P* = 0.017) when considering the entire data set (i.e. without taking into consideration the time of treatment).

NHF1-hTERT samples were also analyzed for the effect of UVC irradiation on fork progression. Table** **2 summarizes the results obtained by measuring the lengths (expressed in arbitrary units) of the CldU-labeled segments of IdU-CldU tracks (Fig.** **3A, patterns 1a and b) to evaluate the movement of replication forks after the cells were exposed to 2.5 J m^−2^ UVC. The aggregated data show that replication fork progression was inhibited (24–41%) at replication units initiated prior to irradiation in early, mid- or late-S phase cells. The shorter track lengths in both sham and UVC-irradiated samples at the 45-min time point are believed to be due to a residual inhibitory effect of aphidicolin.

**Table 2:** Analysis of DNA fork progression in NHF1-hTERT cells pulsed for 10 min with IdU, sham treated or UVC irradiated (2.5 J m^−2^) and pulsed for 20 min with CldU. Aggregate data from 4 independent experiments show the arbitrary length of the CldU-labeled segment of replication units that initiated before irradiation (IdU-CldU tracks)*

	Time in S					
	45 min		3 h		5 h	
	Sham	UVC	Sham	UVC	Sham	UVC
Average	18.7	11.0	31.4	23.9	31.1	18.9
Standard Deviation†	10.1	6.2	14.9	14.3	15.2	14.0
Number Scored	247	210	133	135	67	98
Median	20.2	11.0	30.0	18.6	28.3	17.5
Relative Percent	100	59	100	76	100	61
*P*-value	<0.0001		0.002		<0.0001	

* Replication tracks with the pattern 1a or 1b illustrated in Fig.** **3A (red-green tracks with pattern 3 were not included in this analysis).

† The large standard deviations do not reflect analytical error in the measurement of the track lengths but are due to intrinsic variations in the fork displacement rate 6,31 and the termination of DNA synthesis in some replication units.

### UVC irradiation induces phosphorylation of Chk1 kinase in early and mid S but minimally in late-S phase

**Figure 4:**
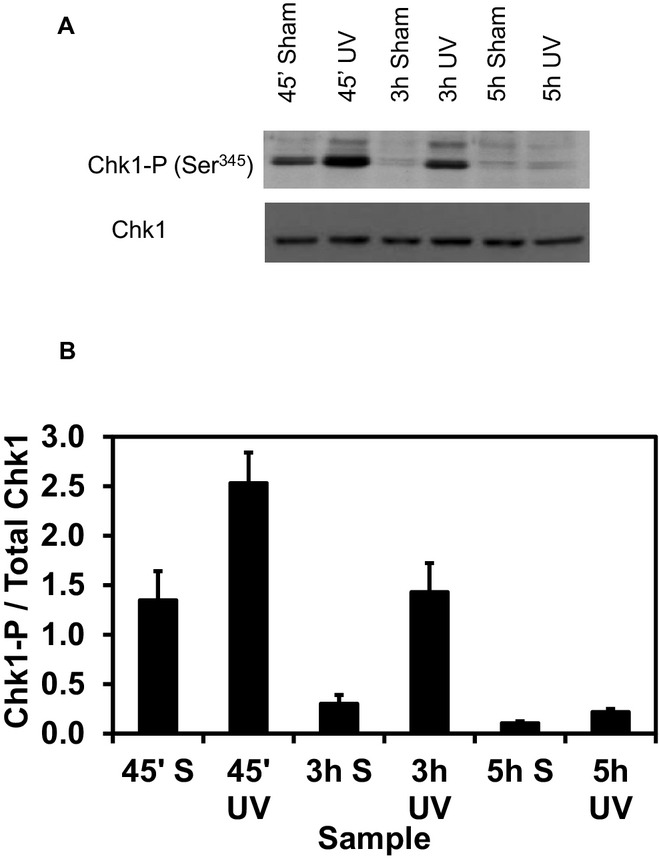
The level of Chk1-P increases when S phase cells are irradiated, but this response is attenuated in late S. (A) Immunoblot for total and phosphorylated Chk1 from a representative synchronization experiment. Equal amounts of protein from NHF1-hTERT that were sham treated or irradiated with UVC (2.5 J m^−2^) at 45 min, 3 h or 5 h into S and incubated for 30 min in reserved medium were separated by SDS-PAGE; the transferred proteins were immunostained for total Chk1 or Chk1-P (Ser^345^). (B) Normalized Chk1-P/total Chk1 values (average ± standard error of the mean) from six independent experiments (see Materials and Methods for details). UVC induced the phosphorylation of Chk1 when the cells were irradiated at 45 min (*P* = 0.0004) and 3 h (*P* = 0.002) but not when the cells were irradiated at 5 h (*P* = 0.44). There was no significant difference between the UVC induction of Chk1-P at 45 min and 3 h (*P* = 0.97), while the response at 5 h significantly differed compared to that at 3 h (*P* = 0.002).

In six independent experiments, including those described above, the level of Chk1-P (Ser^345^) in the sham and irradiated samples was determined by immunoblot analysis. A representative blot is shown in Fig.** **4A; the bar graph in Fig.** **4B includes the average results for the density of the Chk1-P signal normalized to the total Chk1 in the same sample. Noteworthy details are a higher Chk1-P signal in the sham-treated cells at 45 min compared with the other sham samples, and the low signal of phosphorylation of this checkpoint kinase at 5 h in S, even after the cells were exposed to UVC. The first finding can be explained by the strong aphidicolin-dependent inhibition of DNA synthesis in cells entering S phase and the time required for the decay of the induced Chk1-P signal (>1 h). Even with this background, the effect of UVC was clearly manifested by a higher level of Chk1-P. Although the low level of Chk1-P in synchronized cells irradiated at 5 h was unexpected, this level correlates with attenuation of the intra-S checkpoint response of inhibition of replicon initiation described above (Fig.** **3B). Note also that the net increase in the Chk1-P signal induced by UVC treatment (calculated by subtracting the signal detected in sham-treated cultures) was 10-fold higher in early and mid S than late S. NHF10-hTERT cells also displayed a similar net increase in Chk1-P after irradiation at 45 min and 3 h and a much attenuated response at 5 h (results not shown).

The statistical analysis showed that the level of Chk1-P after UVC irradiation was significantly higher (*P* < 0.002) than that observed in the sham-treated samples at 45 min and 3 h but not at 5 h (*P* = 0.44). No significant differences were found between the magnitude of the induction of Chk1 phosphorylation in the early and mid S phase cells. However, the response of late-S phase cells (low UVC-induced phosphorylation of Chk1) was significantly different (*P* < 0.002) from that observed in the mid S phase cells (strong UVC-induced Chk1-P signal).

**Figure 5:**
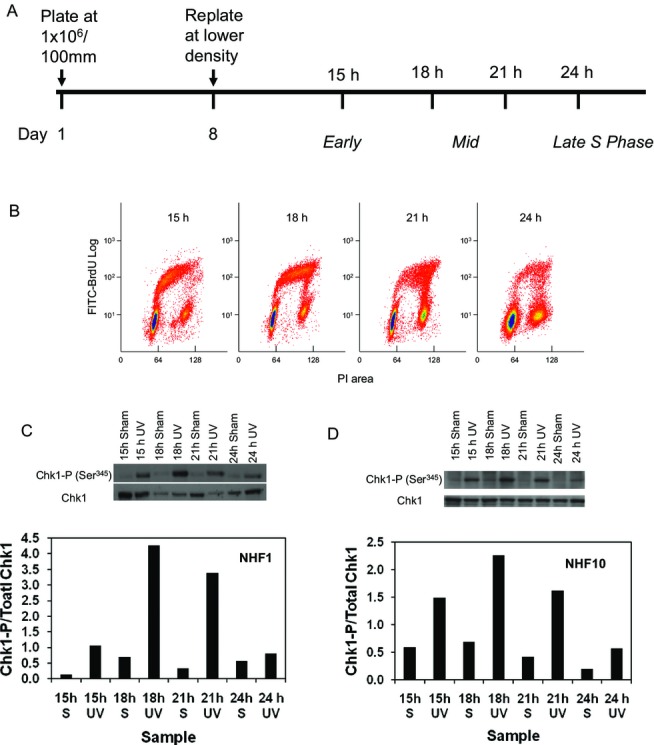
UVC-induced Chk1 phosphorylation is attenuated in late S in cells synchronized only by release from confluence arrest. (A) Schematic drawing of the synchronization protocol. (B) Bivariate flow cytometric analysis of NHF1-hTERT labeled with BrdU for 60 min starting 15, 18, 21 or 24 h after release from confluence arrest. The percentage of cells in S at 15, 18, 21 or 24 h after release from confluence arrest was 44, 41, 23 and 16%, respectively. (C) Equal amounts of protein from NHF1-hTERT cells that were sham treated or irradiated with UVC (2.5 J m^−2^) at 15, 18, 21 or 24 h were separated by SDS-PAGE; the transferred proteins were immunostained for Chk1 and Chk1-P (Ser^345^) kinase. The histogram shows the Chk1-P signal normalized to the total Chk1 at each time point for the sham-treated and irradiated cells. (D) The experiment described in (A–C) was repeated in NHF10-hTERT; the histogram shows the Chk1-P signal normalized to total Chk1 at each time point for sham-treated and irradiated cells. The percentage of cells in S at these time points was 24, 27, 17 and 8%, respectively.

The observation that the sham-treated samples at 45 min into S displayed a higher Chk1-P than the two later time points (Fig.** **4) led us to examine whether fluctuations in the UVC-induced Chk1-P signals could also be detected as normal fibroblasts traversed the S phase using a synchronization protocol that did not include aphidicolin (Fig.** **5A). Although the latter protocol does not yield as tight a cohort of synchronized cells (Fig.** **5B), we detected stronger induction of Chk1 phosphorylation in NHF1-hTERT (Fig.** **5C) and NHF10-hTERT (Fig.** **5D) populations enriched for early and mid S phase cells than late-S phase cells. Together, the data for the two synchronization approaches in NHF originating from two different donors suggest similar patterns of UV-induced Chk1-P in synchronized diploid human fibroblasts. They also suggest that the lower induction of Chk1-P in late-S cells exposed to UVC and concomitant attenuation of radiation-induced inhibition of replicon initiation are stereotypical responses of normal human fibroblasts about to complete the duplication of their genomes.

## Discussion

The responses of S phase cells to DNA damage include (1) passive stalling of active replication forks, (2) maintenance of the stability of stalled replication forks to allow lesion bypass and recovery of DNA replication and (3) slowing of S phase progression by actively inhibiting the initiation of new replicons and reducing the fork progression rate. We used two immortalized lines of normal diploid human fibroblasts (NHF1-hTERT and NHF10-hTERT) to determine how UVC irradiation affects DNA synthesis and checkpoint response in early, mid- and late-S phase fibroblasts. Increases in the levels of Chk1-P (Figs.** **4, 5C–D) and decreases in global DNA synthesis (Fig.** **2) represent endpoints measured in the entire population of cells exposed to UVC at the indicated time windows during progression of the synchronized cells through the S phase. These results track well with those previously measured in log-phase populations of diploid human fibroblasts 18. Asynchronous NHF1-hTERT populations display a well characterized inhibition of replicon initiation by UVC, as measured by changes in distribution of sizes of labeled nascent DNA in alkaline sucrose gradients, which is dependent on the ATR/Chk1 pathway 18,25. Differential immunostaining of DNA replication tracks allows for a better discrimination of new initiation events, but provides information only on those regions of the genome undergoing duplication 7. Hence, it is important to keep this experimental limitation in mind when interpreting the results illustrated in Table** **1 and Fig.** **3B. The effect of UVC on replicon initiation in this case is reflected by the reduction in the representation (percent) of CldU-only tracks relative to the sham-treated, matched control. Such a percentage should not be interpreted as frequency of initiation (which would require the knowledge of the total number of origins of replication that are activated once within each S phase period). The results of new initiation events (CldU-only tracks) (Fig.** **3B, Table** **1, and fork progression rates (Table** **2) reported here indicate that UVC irradiation caused similar responses (type and degree) at the beginning and middle of S phase that were observed in previous studies with log-phase human cells 7,10. However, cells exposed to UVC in late S did not display a strong burst in the Chk1-P signal (Fig.** **4) and did not inhibit origin firing (Fig.** **3B) as efficiently, suggesting an attenuation of the intra-S DNA damage checkpoint response of inhibition of replicon initiation in late-S phase.

Several potential reasons exist why normal human fibroblasts inhibit replicon initiation in early and mid S but not late S. One reason might be fewer active replication forks in late S. A smaller number of active replication forks late in S would decrease the chance of polymerases becoming uncoupled from helicases and generating single-stranded DNA regions, which would be covered by RPA and create the molecular substrate for ATR activation and Chk1 phosphorylation 3,4,26. Comparison of the immunoblot data for Chk1-P (Fig.** **4) and the overall rate of DNA synthesis (Fig.** **2) would support the idea that the low level of Chk1-P being generated late in S is just a consequence of a reduced number of active replication forks encountering UVC-induced DNA damage sites. However, the low level of Chk1-P does not seem to correlate well with the overall rate of DNA replication. For instance, the DNA replication rate in late-S phase is approximately one-third of that observed in mid S, while the Chk1-P signal is one tenth. In early S, replication is two-fold lower than that in mid S, but UVC induces a similar increase in the amount of Chk1-P as observed in mid S. The results reported in Table2 also confirm that UVC induces as strong an inhibition of DNA replication fork progression in early S as in late-S phase. Even though the results reported here do not allow discrimination between the active and passive effects of DNA damage on replication fork progression, we have observed in log-phase human cells that said inhibition is as pronounced after exposure of cells to 1 J m^−2^ UVC as with 2.5 J m^−2^ UVC (P. D. Chastain, M. Cordeiro-Stone, unpublished).

Another potential reason for the lack of a classical checkpoint response late in S *versus* early and mid S is the lack of downstream origins to inhibit late in S. It is postulated that when cells are exposed to DNA damage the origins responsible for the replication of the next tier of chromosomal domains are inhibited, whereas origins that have become refractory to inhibition 5, or are required to ensure that the region experiencing replication stress is completely replicated, do fire 27. In light of this model, the inhibition of origin initiation observed after DNA damage in early and mid S cells could conceivably correspond to cells delaying the firing of origins set to initiate in different chromosomal domains to focus on those that are in the process of being replicated. Along those lines, the lack of origins being inhibited in late S is just a reflection of the absence of chromosomal regions that need to be replicated after the current areas are duplicated.

Another potential reason why origins late in S are refractory to being inhibited is due to Polo-like kinase 1 (Plk1). Plk1 is thought to enable regions that need to finish being replicated after DNA damage to initiate new replication origins by interfering with Chk1 suppression of those origins 28,29. It is unclear why Plk1 does not interfere with origins that are part of the next bank of replicons. It is tempting to speculate that perhaps Plk1 only gains access to actively replicating chromatin and can only interfere with Chk1 at those locations, whereas the origins in the next bank of replicons have a more closed configuration, and thus are not affected by Plk1. Late in S, Plk1 levels are higher than they are in mid and early S, which would further contribute to all late-firing origins being refractory to Chk1-dependent inhibition of initiation 30.

In conclusion, the results reported here suggest that the activation of the intra-S checkpoint is modulated by ATR activation and its phosphorylation of Chk1, but the checkpoint response of inhibiting replicon initiation is also dependent on where the potential targets for inhibition are in the genome (and potentially their chromatin structure). If origins can be differentially regulated to initiate at different times in S phase, then perhaps additional elements of DNA replication (initiation and elongation) might also vary in their degree of sensitivity to inhibition by the DNA damage intra-S checkpoint.

## Abbreviations

ATR: ATM- and Rad3-related protein
BrdU: 5-bromo-2-deoxyuridine
CldU: 5-chloro-2-deoxyuridine
Chk1: checkpoint kinase 1
Chk1-P: phosphorylated checkpoint kinase 1
IdU: 5-iodo-2-deoxyuridine
NHF: normal human fibroblast
RDS: radio-resistant DNA synthesis
UVC: ultraviolet light wavelengths less than 280 nm
